# Phacoemulsification in Nanophthalmic Eye, a Way to Manage Glaucoma: Case Report

**DOI:** 10.1155/2024/2633679

**Published:** 2024-07-01

**Authors:** Dania Bamefleh, Konrad Schargel, Valmore A. Semidey, Faisal A. Altahan, Edward Schargel

**Affiliations:** ^1^ Glaucoma Division King Khaled Eye Specialist Hospital, Riyadh, Saudi Arabia; ^2^ Vitreoretinal Division King Khaled Eye Specialist Hospital, Riyadh, Saudi Arabia; ^3^ College of Medicine King Saud University Medical City, Riyadh, Saudi Arabia; ^4^ College of Medicine Medical University of Varna, Varna, Bulgaria

**Keywords:** closed-angle glaucoma, nanophthalmos, phacoemulsification, short eyes

## Abstract

A rare condition called nanophthalmos causes variable degrees of vision impairment. One may present with nanophthalmos as a hereditary or sporadic condition. There have been documented cases of nanophthalmos treated with bilateral cataract extraction and intraocular lens (IOL) implantation for intractable secondary glaucoma or chronic angle-closure glaucoma. We describe a case of closed-angle glaucoma in a nanophthalmic eye with increased intraocular pressure (IOP) on full medical treatment, along with concurrent drug side effects. As a first surgical procedure, we recommend phacoemulsification of the clear lens + IOL. The challenge in treating nanophthalmic eyes lies in managing the possibility of developing glaucoma in an eye where anatomical conditions make surgery extremely risky. This must be balanced against the advantages of lessening exposure contact in the trabecular meshwork and optimizing the anterior chamber for potential future glaucoma surgery, which can improve the prognosis in these cases. Lastly, it is critical to have a thorough conversation with the patient about the aims, risks, and advantages. The patient's understanding and expectations should also be crystal apparent. The primary objective should always be to enhance the circumstances for the most effective glaucoma therapy, not to perform refractive surgery.

## 1. Introduction

Nanophthalmos is a rare disorder with a varying degree of visual impairment, amblyopia, and a high incidence of angle-closure glaucoma [1]. These very small eyes with high hyperopia commonly do not have gross structural defects.

Nanophthalmos may present as a sporadic or familial disorder with autosomal dominant or recessive inheritance. NNO1 is the first human locus associated with nanophthalmos, or an angle-closure glaucoma phenotype [2]. Five genes (MFRP, TMEM98, PRSS56, BEST1, and CRB1) and two loci have been implicated in familial forms of nanophthalmos [3]. Severe MFRP gene mutations result in recessive nanophthalmos, and it appears that the MFRP gene's embryonic function is essential for the eye to develop fully at birth. This suggests that this gene is necessary for emmetropisation, a complicated process that controls how the eye grows along its axis [4].

A clinical phenotype of simple microphthalmos, nanophthalmos, has short axial length due to shortening of the anterior and posterior segments and thickened sclera. Typical clinical manifestations of nanophthalmos include the small cornea, shallow anterior chamber, and increased lens-to-eye volume ratio, which can increase the risk of closed-angle glaucoma. There are two types of partial microphthalmos: posterior microphthalmos, which has a short axial length because the posterior segment is shorter, and anterior microphthalmos, which has a normal-sized anterior segment and thickened sclera [5]. Relative anterior microphthalmos (RAM) is a condition with short axial length because the anterior segment is shorter than the posterior segment, but the sclera is not thicker. The prevalence of RAM is around 6%. It was associated with small pupils, corneal guttae, glaucoma, and pseudoexfoliation. Intraoperatively, RAM is associated with overall surgical difficulty because there is less working space and a higher risk of uveal trauma, Descemet's detachment, and posterior capsule rupture. Postoperatively, RAM is associated with transient corneal oedema [6].

We show a case of closed-angle glaucoma in a nanophthalmic eye with high intraocular pressure (IOP) that was getting full medical treatment but having side effects from the medications. As the first surgery, we suggest phacoemulsification of the clear lens plus intraocular lens (IOL).

## 2. Case Report

**Figure 1:**
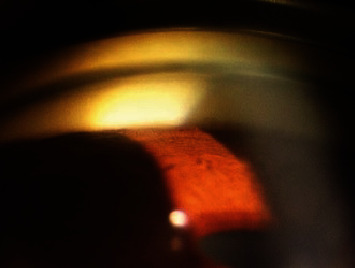
Gonioscopy view of the 6 o'clock. Close angle. All quadrants were the same.

**Figure 2:**
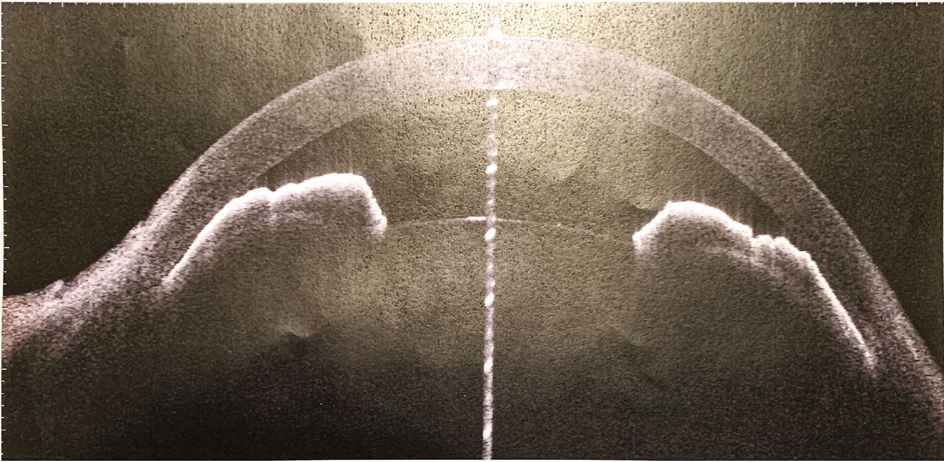
Anterior segment OCT: iridocorneal touch is evident in the periphery, and a centrally reduced space in the anterior chamber can be seen.

**Figure 3:**
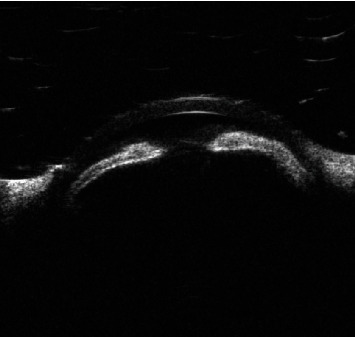
UBM preoperative evaluation showing close angle.

**Figure 4:**
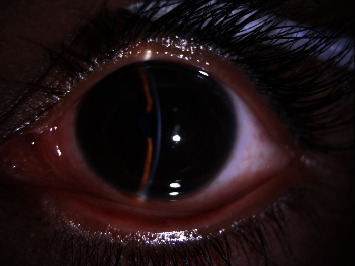
Anterior segment view, shallow anterior chamber, noticeable in the center, before surgery.

**Figure 5:**
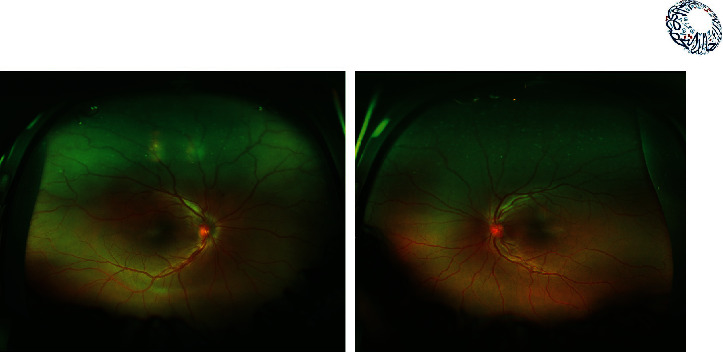
Fundus photo. Optic nerve looks crowded.

**Figure 6:**
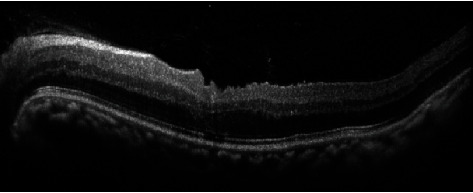
Posterior segment OCT, macular folds.

A 26-year-old lady presented with the best corrected visual acuity (BCVA) of Log Mar 0.3 OU, to +14.00 D (diopters) in the right eye (OD) and +14.75 D in the left eye (OS) of refractive error. The Zeiss IOLMaster 700® optical biometer test showed that the axial lengths were 15.65 mm OD and 15.37 mm OS, and the keratometry was OD K1 49.27 at 147° and K2 50.45 at 57 with astigmatism of −1.18 at 147; the net anterior chamber was 2.05 mm and OS K1 49.71 at 16° and K2 51.45 at 106° with astigmatism of −1.74 at 16°. The ACD was 2.23 mm. With a white-to-white (WTW) OD of 10.58 mm and OS of 10.56 mm, her IOP has fluctuated from as high as 30 down to 12 mmHg. Her most recent readings were 14 mmHg OD and 10 mmHg OS. She is taking brimonidine, timolol (Combigan®, Allergan), bimatoprost (Lumigan®, Allergan), and 250 mg of acetazolamide twice a day. The patient was experiencing systemic and local side effects related to the medications used. The patient had closed angles (Figure 1). We used an Nd-YAG laser for a peripheral iridotomy (Lumenis Selecta Duet YAG/SLT Laser) to fix this. After this, she developed some peripheral anterior synechiae (PAS). The Heidelberg Spectralis® optical coherence tomography (OCT) (Figure 2) and the Quantel Aviso ultrasound biomicroscopy (UBM) ophthalmic ultrasound (Figure 3) showed that the iris pushed forward and had 360° of closed angles. The anterior segment picture correlates with the test, showing a shallow anterior chamber even in the center (Figure 4). Fundus examination (Figure 5) showed crowded discs with mild horizontal macular folds clinically, and OCT (Heidelberg Spectralis®) (Figure 6) evidenced wrinkling of the inner retinal layers consistent with the mild macular fold. In contrast, the outer retinal layers appeared unaltered.

**Figure 7:**
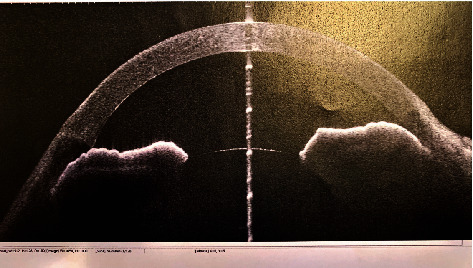
The anterior segment OCT, which shows that the iridocorneal touch decreases the angle, has not fully opened. The anterior chamber's increased depth can be seen.

We suggested taking out the clear lens and implanting an IOL to make the anterior chamber deeper (Figure 7) and reducing the iridocorneal touch so that the IOP could be better controlled. A deep discussion with the patient was conducted, carefully explaining the risk of choroidal effusion and expulsive hemorrhages as well as the benefits, which mainly included controlling the IOP and reducing medication, avoiding further damage to the trabecular meshwork tissue by the iridocorneal contact, and insisting that this was not a refractive surgery purpose. The right eye was operated on first.

One day before the surgery, we used Mannitol 20% IV at a dose of 1 g/kg of weight. Mannitol IV was repeated 2 h before the surgery at the same dose. Based on her previous general medical state, especially renal function evaluation by internal medicine, Mannitol was used for vitreous dehydration and creating more space. As mentioned before, the patient was already on full topical medication and systemic acetazolamide preoperatively. We choose to work through two paracenteses, made with an MVR 23 G (Mani®), in the right eye, 11 o'clock and 7 o'clock, leaving 9 o'clock for the main incision, with a keratome of 2.2 mm (Mani®), which was made later for the phacoaspiration and lens insertion. The 1 and 5 o'clock positions were used in the left eye, leaving the 3 o'clock position for the main incision. Using two small paracenteses (23 G) aims to keep the eye pressurized at all moments, avoiding decompression and maintaining the space to facilitate the capsulorhexis, as the vitreous was partially dehydrated with the Mannitol. We used a combination of dispersive and cohesive ophthalmic viscoelastic devices (OVDs) to maintain the space Discovisc® (Alcon) and Provisc® (Alcon). As it was a clear lens extraction, we mainly used phacoaspiration with a Centurion® phacoemulsification machine (Alcon®), and almost no emulsification or torsional energy was needed.

We calculated the IOL selection using an average of the power between the Hoffer Q, Barret, and Haigis formulas. We aimed for emmetropia and utilized a customized IOL Bi-Flex HL (677AD) from Medicontur® (50 and 48 D for the right and left eye, respectively). This is a single-piece, foldable acrylic aspheric hydrophilic and monofocal posterior chamber IOL with UV blocker. The IOL is non-preloaded and must be manually loaded into a compatible injector, inserted through a 2.4 mm enlargement of the phacoemulsification original main incision.

Viscogonioplasty under indirect view with Gonioscopic lens Mori® from Ocular Instruments was done using Provisc® Alcon and mechanically trying to separate the iris's apposition against the cornea. Through the main incision and the two paracenteses, reaching 200° to 220°.

The main incision was secured with one stitch, nylon 10-0 (Ethicon®).

**Figure 8:**
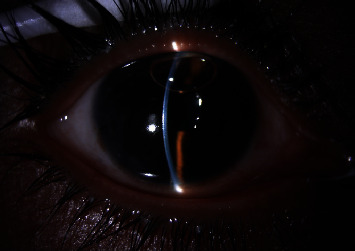
Twenty-four-hour postoperative increase in depth in the anterior chamber, with a bubble of air to keep the IOL in place.

Viscoelastic was removed with bimanual handpieces through the paracenteses. Acetylcholine (Miochol®) was used, and air bubbles were left in the anterior chamber to ensure the IOL remained in the bag for the first 24 to 36 h (Figure 8). At the end of the procedure, intracameral cefuroxime 0.1 mL was administered, and acetazolamide 250 mg twice a day for the first 24 h was maintained.

The follow-up visits were the next day, 1 week, 1 month, 3 months, 6 months, and 1 year. Because of the high risk of developing glaucoma, the patient is still under observation in the Glaucoma Division of King Khaled Eye Specialist Hospital, Riyadh, Saudi Arabia.

Visual acuity, autorefraction, applanation tonometry, anterior and posterior segment OCT, fundus photography, and anterior segment photography were performed at all visits, but in the 1-week visit, only VA, tonometry, slit lamp examination, and posterior pole examination were done.

At the 1-month postoperative visit, refraction was performed in the first eye (OD) to adjust the calculation for the second eye IOL. Final refraction was performed in both eyes 12 weeks after the second eye was operated on. The final refractive error was +1.25 OD Plano OS, with a final visual acuity of 0.18 Log Mar OD and 0.48 Log Mar OS.

**Figure 9:**
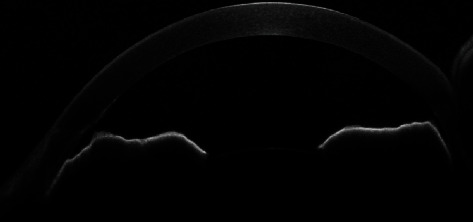
AS-OCT 1-year postoperative iridocorneal touch decreases, and the angle is narrow but not as close as before surgery.

One year after the lens extraction, the eyes remained quiet, visual acuity was stable without correction, using reading glasses, and the average IOP was 16 mmHg with one preservative-free medication, with no signs of glaucoma progression. Angles remained narrow (Figure 9), but no acute crises were reported. However, we have noticed some posterior capsular opacification development, which might explain the slight decrease in final visual acuity. Still, the patient is satisfied because the dependence on glasses is mainly for reading.

## 3. Discussion

Bilateral cataract extraction and IOL implantation have been used for patients with persistent secondary glaucoma or chronic angle-closure glaucoma related to nanophthalmos.

Cataract surgery deepened and broadened the anterior chamber angle in eight patients out of 11 from the retrospective, noncomparative, interventional case series of Deng et al. [7].

We present a case of nanophthalmic eyes that were followed because of a close angle, with fluctuations of the IOP, that did not respond to complete medical treatment plus oral acetazolamide nor to iridotomies. After a lengthy discussion with the patient on the risks and benefits, the patient underwent a clear lens extraction, which had an excellent outcome.

The B-scan and UBM revealed increased retinal-choroidal-scleral thickness (average mean 241 *μ*C, peripherical 100 *μ*C, and posteriorly 300 *μ*C) in nanophthalmic eyes, which is more evident if those eyes have uveal effusion that can range from 500 to 1000 *μ*C. The characteristic of these eyes is the regular distribution of the thickness [8–11]. In hyperopic eyes and those at risk for narrow-angle glaucoma or undiagnosed nanophthalmos, echography and UBM should be utilized to measure retinal-choroidal-scleral thickness. An increase in thickness also increases the risk of uveal effusion [12].

The sclera is centrally essential to vision. It maintains refractive status with the cornea and must also provide stable mechanical support to vulnerable internal ocular structures such as the retina and optic nerve head. Moreover, it must achieve this under complex, dynamic loading conditions imposed by eye movements and fluid pressures [13].

Different methods, like swept-source OCT (SS-OCT), can measure the scleral thickness. This method allows imaging of the posterior sclera, including the subfoveal and optic nerve head regions. It has been used to measure scleral and laminar thicknesses in patients with glaucoma. SS-OCT showed good reproducibility in measuring scleral thickness and detected differences in thickness between normal eyes and different glaucoma types [12–14].

UBM has been used to measure scleral thickness [15] in patients with uveal effusion syndrome (UES) [16]. It is a functional adjunctive test for managing this condition. Compared to magnetic resonance imaging (MRI), UBM may be a more accurate and precise method of measuring scleral thickness [17].

A thicker sclera has been associated with a higher risk of UES [18, 19]. UES is marked by spontaneous ciliochoroidal detachment, and it is thought that the thick sclera presses on the vortex veins, which causes fluid to build up in the spaces under the retina and on top of the choroidal layer [20]. Nanophthalmos, a condition characterized by thick sclera, among other characteristics, is particularly prone to developing UES. However, there have been cases of UES in non-nanophthalmic eyes with histologically normal sclera [21]. There are no guidelines we could suggest on which thickness will be the right one to do the partial sclerotomies to relax the compression, as some studies showed that using sclerotomies will not prevent UES. Based on our experience, 500 *μ*C is the limit; under 500 *μ*C and no other risk factor, we do the cataract surgery without sclerotomies; over 500 *μ*C, we do the sclerotomies in combination with the lens extraction [22].

In healthy eyes, cataract extraction results in an enlargement of Schlemm's canal, which may reduce IOP [23, 24]. In the nanophthalmic eye, increased compression may be alleviated by removing the lens, lowering IOP.

According to some studies, in eyes with an anatomically shallow peripheral anterior chamber but no PAS at the time of diagnosis, prolonged iridotrabecular contact contributed to the development of PAS over 7 years of follow-up in almost half of the cases [25].

In primary closed-angle disease (PACD), the morphological features of the lens have been studied, and it has been found that the lens plays a vital role in the anatomical shallow anterior chamber. These eyes show a statistically significant difference versus matched controls in terms of anterior chamber depth, axial length, iridotrabecular contact index, lens vault, and lens thickness. These findings suggest that the crystalline lens morphologic features may contribute significantly to the development of PACD [26, 27]. The reduction in IOP appears to be more related to the anterior vault of the lens [28].

In primary angle-closure glaucoma (PACG) patients, the clinical presentation is not always clear; these patients might only present with PAS but no acute attacks. It was looked at whether there was a link between the size of the PAS and how bad the visual field defects were in people with PACG. It found a statistically significant link between the size of the PAS and the severity of visual field damage in PACG patients, especially those who had never had an acute attack [29].

After cataract surgery, the widening of the angle in the eye is associated with a more significant drop in IOP. This reduction in IOP is more critical in eyes with narrow angles than those with open angles. Phacoemulsification is an effective method for treating narrow angles [30]. The angle in our case opened, but due to the eye being too short, the contact between the iris and cornea persisted, although not to the same extent. This may explain the moderate decrease in IOP, managed with a single preservative-free drop.

Management of glaucoma in the nanophthalmic eye remains a challenge. In a retrospective study of 28 patients, trabeculectomy with MMC and inferior sclerotomy procedures were effective and safe for glaucoma control in patients with nanophthalmos, but uveal effusion was a significant problem, and cataract surgery is expected in the long term [31].

A combination of pars plana lensectomy in nanophthalmic eyes with glaucoma in a small series of 21 eyes was effective in lowering the IOP from 47.4 ± 5.7 to 18.6 ± 3.6 mmHg and antiglaucoma medication from 5 to 0. IOP control was achieved by eliminating the pupillary block and deepening the anterior chamber. This surgical procedure proved safe for the eyes, but there was a potential risk of vision-threatening complications [32].

Phacoemulsification (closed intraocular microsurgery) has enhanced cataract management in nanophthalmic eyes, with endothelial cell loss comparable to normal eyes. Factors primarily impacting endothelial cell density are an anterior chamber depth below 2.5 mm and advanced age [33]. The patient's condition involves a shallower anterior chamber, but due to their young age, no impairment was observed in endothelial cell count after 1 year.

The refractive predictability and postoperative outcome in extreme eyes such as nanophthalmos are poorer than those with RAM or standard control eyes [34].

A case in the literature describes custom-made IOLs with powers of +56.0 and +58.0 D from Human Optics AG, Erlangen, Germany. The authors encountered a similar challenge when calculating the IOL power for emmetropia and, like us, utilized the mean value of the formulas [35]. We needed to select an IOL that would result in a favorable final refractive error for a small eyeball. We utilized the Hoffer Q, Barrett, and Haigis formulas with the Zeiss IOL Master® 700 to determine the IOL, which was more effective in comparable scenarios according to most research [36].

Our case had an uneventful postoperative course, with controlled IOP with medication without the need for filtration surgery or vitrectomy.

We think that nanophthalmic eyes represent a real surgical and clinical challenge; potential glaucoma that can develop over time and the anatomical conditions that will make any surgery of high risk need to be balanced with the possibility of reducing the exposure contact in the trabecular meshwork and increasing the space in the anterior chamber for possible future glaucoma surgery, which will improve the outcome in these cases.

Finally, a complete discussion with the patient on goals, risks, and benefits is essential, and patient comprehension and expectations should be clear. The main goal should never be refractive surgery; the main goal is to improve the conditions for the best management of glaucoma.

## Data Availability

All data generated or analyzed during this study are included in this article. Further inquiries can be directed to the corresponding author.
